# Cognitive training for the prevention of skill decay in temporarily non-performing orthopedic surgeons

**DOI:** 10.1080/17453674.2020.1771520

**Published:** 2020-06-05

**Authors:** Robi Kelc, Matjaz Vogrin, Janja Kelc

**Affiliations:** a Department of Orthopedic Surgery, University Medical Center Maribor;; b Institute of Sports Medicine, FIFA Medical Center of Excellence, Faculty of Medicine, University of Maribor;; c Department of Psychiatry, University Medical Center Maribor, Slovenia

## Abstract

Surgical tasks are prone to skill decay. During unprecedented circumstances, such as an epidemic, personal illness, or injury, orthopedic surgeons may not be performing surgical procedures for an uncertain period of time. While not being able to execute regular surgical tasks or use surgical simulators, skill decay can be prevented with regular mental practice, using a scientifically proven skill acquisition and retaining tool. This paper describes different theories on cognitive training answering the question on how it works and offers a brief review of its application in surgery. Additionally, practical recommendations are proposed for performing mental training while not performing surgical procedures.

This paper has previously been shared with the Slovenian Orthopedic Association as a blog post.

Surgical tasks combine many gross and fine motor actions in a strict time frame, demanding a high degree of accuracy. As such, surgical skills usually present with a flat learning curve, especially in the case of minimally invasive operations. However, once acquired, surgical skills are prone to decay, especially after a period of not practicing (Sonnadara et al. 2012, Routt et al. 2015). Other important risk factors affecting decay are time pressure and the quality of the job performed (Wisher et al. 1999), both being vital components of surgical performance.

During special circumstances, such as an epidemic (e.g., COVID-19), surgeons may be relocated to working environments away from their field of expertise, thereby not performing surgical procedures for a period of time. Similarly, an interval of not practicing is inevitable in the case of a surgeon’s illness or injury. While not being able to perform regular surgical tasks or use surgical simulators, the prevention of skill decay can be achieved through regular mental practice, using a scientifically proven skill acquisition and retaining tool.

Mental practice or imagery, a form of cognitive training, is a symbolic rehearsal of a physical activity in the absence of any gross muscular movements (Richardson 1967). It is widely implemented in sports where it has long been used with success in enhancing the performance of elite athletes (Martin et al. 1999) and in certain other areas, such as aviation (Fornette et al. 2012), professional music (Bernardi et al. 2013), and surgery (Wallace et al. 2017). All these fields share crucial similarities, such as the importance of technical skills, performance under stressful conditions, and aiming for perfection without making mistakes. Mental practice improves a variety of different motor skills in sports, as well as acquisition, physical strength (Sevdalis et al. 2013), and technique performance (Surburg 1968), hence its application in surgery is not only scientifically justified but also common sense.

It is believed that experienced subjects may benefit more from mental practice on physical tasks because of the requisite schematic knowledge to imagine an accurate and a precise outcome associated with the imagined performance (Posner 1989). For example, experienced athletes have better visualizing abilities and employ more structured mental practice sessions in comparison with novices (Feltz and Landers 2007). However, mental practice has also recently gained in popularity with novice surgeons, surgical trainees, and medical students learning new surgical techniques (Hall 2002, Sanders et al. 2004). Cognition, integration, and automation are typical steps in learning new surgical skills (Hamdorf and Hall 2000), the first two being most decay-susceptible and therefore a subject of interest for mental practice as a retaining tool.

This article provides a brief theoretical background on cognitive training, followed by its application in surgery and surgical education. Finally, recommendations are provided for novice and expert surgeons for performing cognitive training while not performing surgery for a long or uncertain period of time.

## Theoretical basis for cognitive training

The motor system has been hypothesized to be part of a cognitive network including a variety of psychological activities. During cognitive training and real-life motor tasks, similar neural paths are being activated. In musicians, a close relationship between motor imagery and motor action has been described, for example changes in corticospinal activity with the same muscles involved in both circumstances (Fadiga et al. 1999).

There are different theories on why mental practice improves motor skills (Vealey and Walter 1993). The psychoneuromuscular theory proposes that mental training causes activation pattern of muscles similar to actual movements (Jacobson 1931). The symbolic learning theory postulates that symbols are coding the sequence of movements (Sackett 1934). The repetition of symbolic components of the movement pattern facilitates execution of an actual motor pattern (Frank et al. 2014). A more recent theory suggests that motor imagery and motor performance are functionally equivalent, thereby suggesting that in both the same underlying neural structures and mechanisms are involved (Jeannerod 1994).

## Cognitive training in surgery

Surgery as a medical specialty containing complex psychomotor and cognitive tasks is without any doubt subject to skill decay when tasks are not being performed for a certain period (Arthur et al. 1998). It is not only a question of one’s interest but is also a surgeon’s duty to master the skills and retain them. Novice surgeons and surgical trainees usually find themselves in cognitive and integrational phases of learning, thus being especially vulnerable to the decay of their skills, which can happen after a short retention interval (Sugihara et al. 2018). On such occasions, cognitive training can be of critical importance as it has been proven to enhance knowledge of a procedure, flow of an operation, and preparedness for the task (Komesu et al. 2009).

There are numerous cognitive training techniques. At a novice level, cognitive task analysis (CTA) training has been shown to be the most effective, a method by which experts are used to construct a teaching program for novices through intuitive knowledge and thought processes (Tofel-Grehl and Feldon 2013, Wingfield et al. 2015). CTA has recently been proven to be an effective technique in hip arthroplasty, where its use resulted in shorter procedure time, decrease in the number of errors, and increase in accuracy of acetabular cup orientation (Logishetty et al. 2020). However, CTA requires a mentor to be present, which is potentially an inaccessible option in some specific situations. In this case, other techniques of cognitive training, such as external observative and subvocal training, are also potentially useful. In the former, a surgeon is an observer of a skill that is to be learned whereas in the latter a visual image is being called up by a surgeon through external or self-talk (Immenroth et al. 2007).

Cognitive training is not beneficial for novice surgeons only. The most experienced surgeons report going over the procedures in “their mind’s eye” and consider mental readiness the most important type of preparation, followed by technical and physical readiness (Sanders et al. 2004). It has been shown that in experts not only are the same regions of the brain being engaged during visual imagery as in novices, but also additional regions are recruited suggesting that the pattern of activation moves from frontal parts at the beginning of the process to posterior parts responsible for retrieval of domain-specific knowledge around the final expertise stage (Bilalić et al. 2012). While cognitive specific skills tend to be the focus of novice subjects who are learning specific movements, cognitive general skills are usually used more by experts who link the skills together. Additionally, experts often use motivational and arousal techniques to enhance overall performance by setting specific goals and managing stress and relaxation (Mace et al. 1986, MacIntyre et al. 2002).

In a review by Wallace et al. (2017) limitations of some studies evaluating cognitive training in surgical education have been identified, like small sample sizes, being focused only on short-term effectiveness and lacking psychological mechanisms that underlie their efficacy. However, they concluded that cognitive training is to be integrated as a training tool for surgeons.

## Recommendations for surgeons

Cognitive training in combination with physical training impacts performance to a greater extent than physical training only. In the absence of specific elective surgical procedures, cognitive training is the only skill acquisition and retaining tool that can and should be used constantly. The literature suggests that mental practice should be brief and focused and should optimally be carried out for 20 minutes in a single session, since extended mental practice may lead to loss of concentration (Corbin 1972, Driskell et al. 1994).

## Training by observation

For mental practice to be effective, the subject must be familiar with the surgical procedure prior to the imagery session (Driskell et al. 1994). It is not useful for one to mentally practice a complex procedure if the individual is not familiar with the procedure and its flow. In such a situation, external observational training should be performed first. This could be realized through observation of recordings from trusted sources (i.e., VuMedi, clips from industrial educational websites, YouTube with well-known surgeons performing, etc.), surgical technique guides, and textbooks. Observing a recording can be followed only by listening to its audio component and visualizing the procedure rather than watching it.

## Subvocal imagery

Subvocalization is a natural process of internal speech typically undertaken during reading. In terms of surgical training, visual images are recalled by an internal self-talk (Immenroth et al. 2007). This type of training represents not only a possible next step in external observative training but also a practical way of performing mental imagery training for more experienced surgeons. Novice surgeons especially should pay attention to every detail in a systematic manner (i.e., patient positioning before shoulder arthroscopy, trocar insertion, opening of the water inflow valve, rotating the optics, identify intra-articular structures, etc.), whereas more experienced surgeons can focus more on a specific difficult step (i.e., placing stitches in a cuff tear and placing a screw in the correct position).

## Ideomotoric training

The ideomotoric training technique implies the movement patterns imagined several times, visualized and verbalized by a highly trained expert to facilitate a procedure execution (Khalmanskikh 2016). The subject visualizes movements from an inner perspective, imagining him- or herself performing the procedure (Immenroth et al. 2007). To include motivational and arousal components of cognitive training, the sensory aspects of the procedures should be included as far as possible. One should recall the atmosphere in the operating theatre such as the team, temperature, light, and even background music and the sounds produced by the anesthetic device. Furthermore, the trainee should try to feel the tactile properties of instruments and tissues (i.e., visualize the polydioxanone suture and how tricky it is to catch it with a tissue grasper in a narrow space, or the challenging passage of the endobutton in cortical ACL fixation). Additionally, potential intraoperative complications and their management (i.e., displacement of implants after using all-internal meniscal devices or suture anchor pull-out after tying a knot in rotator cuff repair) should not be left out.

## Conclusion

In extraordinary conditions, specific orthopedic surgical skills are prone to decay in the case of nonperformance. Cognitive training has been shown to be an effective tool for performance improvement when performed alone or in combination with motor training under ordinary circumstances. In the case of limited access to actual performance, mental practice seems to be reasonable if not mandatory for both novice and experienced surgeons. Not only would such training prevent surgical skill decay but it would probably also lower the anticipatory anxiety level on returning to the operating theatre. 

## References

[CIT0001] Arthur J, Bennett W, Stanush P L, McNelly T L. Factors that influence skill decay and retention: a quantitative review and analysis. Human Performance 1998; 11(1): 57–101.

[CIT0002] Bernardi N F, De Buglio M, Trimarchi P D, Chielli A, Bricolo E. Mental practice promotes motor anticipation: evidence from skilled music performance. Front Hum Neurosci 2013; 7. 10.3389/fnhum.2013.00451 PMC3747442 23970859

[CIT0003] Bilalić M, Turella L, Campitelli G, Erb M, Grodd W. Expertise modulates the neural basis of context dependent recognition of objects and their relations. Hum Brain Mapp 2012; 33(11): 2728–40. 21998070 10.1002/hbm.21396 PMC6870017

[CIT0004] Corbin C B. Mental practice. In: WP Morgan, editor. Erogenic aids and muscular performance. San Diego, CA: Academic Press; 1972. p. 93–118.

[CIT0005] Driskell J E, Copper C, Moran A. Does mental practice enhance performance? J Appl Psychol 1994; 79(4): 481–92.

[CIT0006] Fadiga L, Buccino G, Craighero L, Fogassi L, Gallese V, Pavesi G. Corticospinal excitability is specifically modulated by motor imagery: a magnetic stimulation study. Neuropsychologia 1999; 37(2): 147–58. 10080372 10.1016/s0028-3932(98)00089-x

[CIT0007] Feltz D, Landers D. The effects of mental practice on motor skill learning and performance: a meta-analysis. J Sports Psychol 2007; 5: 25–57.

[CIT0008] Fornette M-P, Bardel M-H, Lefrançois C, Fradin J, Massioui F E, Amalberti R. Cognitive-adaptation training for improving performance and stress management of Air Force pilots. Int J Aviat Psychol 2012; 22(3): 203–23.

[CIT0009] Frank C, Land W M, Popp C, Schack T. Mental representation and mental practice: experimental investigation on the functional links between motor memory and motor imagery. PLoS ONE 2014; 9(4): e95175. 24743576 10.1371/journal.pone.0095175 PMC3990621

[CIT0010] Hall J C. Imagery practice and the development of surgical skills. Am J Surg 2002; 184(5): 465–70. 12433615 10.1016/s0002-9610(02)01007-3

[CIT0011] Hamdorf J M, Hall J C. Acquiring surgical skills. Br J Surg 2000; 87(1): 28–37. 10606907 10.1046/j.1365-2168.2000.01327.x

[CIT0012] Immenroth M, Bürger T, Brenner J, Nagelschmidt M, Eberspächer H, Troidl H. Mental training in surgical education: a randomized controlled trial. Ann Surg 2007; 245(3): 385–91. 17435545 10.1097/01.sla.0000251575.95171.b3 PMC1877007

[CIT0013] Jacobson E. Electrical measurements of neuromuscular states during mental activities, V: Variation of specific muscles contracting during imagination. Am J Physiol 1931; 96: 115–21.

[CIT0014] Jeannerod M. The representing brain: neural correlates of motor intention and imagery. Behav Brain Sci 1994; 17(2): 187–245.

[CIT0015] Khalmanskikh A V. Ideomotor training to improve shooting skills in elite biathlon 2016. Available at: http://www.teoriya.ru/ru/node/5815. Accessed March 29, 2020.

[CIT0016] Komesu Y, Urwitz-Lane R, Ozel B, Lukban J, Kahn M, Muir T, Fenner D, Rogers R. Does mental imagery prior to cystoscopy make a difference? A randomized controlled trial. Am J Obstet Gynecol 2009; 201(2): 218.e1–9. 19481728 10.1016/j.ajog.2009.04.008 PMC3623945

[CIT0017] Logishetty K, Gofton W T, Rudran B, Beaulé P E, Gupte C M, Cobb J P. A multicenter randomized controlled trial evaluating the effectiveness of cognitive training for anterior approach total hip arthroplasty. J Bone Joint Surg Am 2020; 102(2): e7. 31567674 10.2106/JBJS.19.00121

[CIT0018] Mace R D, Carroll D, Eastman C. Effects of stress inoculation training on self-report, behavioral and psychophysiological reactions to abseiling. J Sports Sci 1986; 4(3): 229–36. 3586115 10.1080/02640418608732121

[CIT0019] MacIntyre T, Moran A, Jennings D J. Is controllability of imagery related to canoe-slalom performance? Percept Mot Skills 2002; 94(3 Pt 2): 1245–50. 12186246 10.2466/pms.2002.94.3c.1245

[CIT0020] Martin K A, Moritz S E, Hall C R. Imagery use in sport: a literature review and applied model. Sport Psychol 1999; 13(3): 245–68.

[CIT0021] Posner M. Foundations of cognitive science. J Hist Behav Sci 1989; 27(3): 242–7.

[CIT0022] Richardson A. Mental practice: a review and discussion. Res Q 1967; 38: 95–107. 5338007

[CIT0023] Routt E, Mansouri Y, de Moll E H, Bernstein D M, Bernardo S G, Levitt J. Teaching the simple suture to medical students for long-term retention of skill. JAMA Dermatol 2015; 151(7): 761–5. 25785695 10.1001/jamadermatol.2015.118

[CIT0024] Sackett R S. The influence of symbolic rehearsal upon the retention of a maze habit. J Gen Psychol 1934; 10: 376–98.

[CIT0025] Sanders C W, Sadoski M, Bramson R, Wiprud R, Van Walsum K. Comparing the effects of physical practice and mental imagery rehearsal on learning basic surgical skills by medical students. Am J Obstet Gynecol 2004; 191(5): 1811–14. 15547570 10.1016/j.ajog.2004.07.075

[CIT0026] Sevdalis N, Moran A, Arora S. Mental imagery and mental practice applications in surgery: state of the art and future directions. In: Lacey S, Lawson R, editors. Multisensory imagery. New York: Springer-Verlag; 2013.

[CIT0027] Sonnadara R R, Garbedian S, Safir O, Nousiainen M, Alman B, Ferguson P, Kraemer W, Reznick R. Orthopaedic Boot Camp II: examining the retention rates of an intensive surgical skills course. Surgery 2012; 151(6): 803–7. 22652121 10.1016/j.surg.2012.03.017

[CIT0028] Sugihara T, Yasunaga M, Matsui H, Ishikawa A, Fujimura T, Fukuhara H, Fushimi K, Homma Y, Kume H. A skill degradation in laparoscopic surgery after a long absence: assessment based on nephrectomy case. Mini-invasive Surg 2018; 2(11).

[CIT0029] Surburg P R. Audio, visual, and audio-visual instruction with mental practice in developing the forehand tennis drive. Res Q 1968; 39(3): 728–34. 4177259

[CIT0030] Tofel-Grehl C, Feldon D. Cognitive task analysis-based training: a metaanalysis of studies. J Cogn Eng Decis Mak 2013; 7(3): 293–304.

[CIT0031] Vealey R S, Walter S M. Imagery training for performance enhancement and personal development. In: Williams JM, editor. Applied sport psychology: personal growth to peak performance. 2nd ed. Mountain View, CA: McGraw Hill; 1993. p 200–24.

[CIT0032] Wallace L, Raison N, Ghumman F, Moran A, Dasgupta P, Ahmed K. Cognitive training: how can it be adapted for surgical education? Surgeon 2017; 15(4): 231–9. 27658665 10.1016/j.surge.2016.08.003

[CIT0033] Wingfield L R, Kulendran M, Chow A, Nehme J, Purkayastha S. Cognitive task analysis: bringing olympic athlete style training to surgical education. Surg Innov 2015; 22(4): 406–17. 25392150 10.1177/1553350614556364

[CIT0034] Wisher R, Sabol M A, Ellis J, Ellis K. Staying sharp: retention of military knowledge and skills. For Belvoir, VA: US Army Research Institute; 1999.

